# Medical student experience with robot-assisted surgery after limited laparoscopy exposure

**DOI:** 10.1007/s11701-020-01129-9

**Published:** 2020-07-23

**Authors:** Nasit Vurgun, Tanawat Vongsurbchart, Aneta Myszka, Piotr Richter, Tomasz Rogula

**Affiliations:** 1 1st Department of Surgery, Jagiellonian University Medical College, ul. Jakubowskiego 2, 30-688 Krakow, Poland; 2 Department of Surgery, Case Western Reserve University, Cleveland, USA

**Keywords:** Laparoscopy, Robotic surgery, Surgical education, Robotic skills, Performance

## Abstract

The purpose of the study was to evaluate the objective and subjective experience of medical students completing robotic surgery tasks after limited laparoscopy exposure. Twenty-three medical students without previous laparoscopy and robotic surgery experience self-enrolled into 0 min (*n* = 11), 20 min (*n* = 6), and 40 min (*n* = 6) laparoscopy training groups. Subjects completed rope passing and ball placement tasks on a laparoscopy trainer before repeating similar tasks on the Senhance Surgical System, a robot-assisted digital laparoscopy device. Videos were recorded to evaluate objective measures including time, completion rate, clutch use, out of view instruments, ball drops, and manual adjustments. The NASA-TLX survey was administered to assess subjective experience using workload and task demand measures. There were no statistically significant differences in objective performance between the groups (*p* > 0.05). Subjects who completed laparoscopy training reported higher workloads, but these differences were not statistically significant (*p* > 0.05). NASA-TLX workload was correlated with time performance on Pearson and Spearman tests (*r* = 0.623, rho = 0.681, *p* < 0.01). Initial experience of medical students with robot-assisted surgery did not differ significantly after limited laparoscopy exposure.

## Introduction

Surgical robots have transformed the landscape of minimally invasive surgery by enabling surgeons to operate with greater amounts of control, comfort, and capability [1]. Haptic feedback, movement scaling, tremor filtering, 3D high-definition vision, camera integration with eye-tracking, and remote operation from a seated console are examples of features that may be found on robot-assisted surgical devices [2]. Compared to open surgery, robotic surgery has demonstrated improved clinical outcomes, including reduced blood loss, transfusion rate, length of hospital stay, and 30-days complication rate after various surgical procedures [3]. Compared to conventional laparoscopy, robot-assisted procedures have similar oncological outcomes and complication rates, although functional outcomes may be superior for urologic, gynecologic and colorectal procedures [4–6]. Higher cost and longer operative times remain a challenge, but many believe that robotic techniques will continue to grow and improve at an exponential rate [7]. Consistent with this optimism are observations showing a shift from laparoscopy to robotic techniques in recent years [8]. If market competition can lower costs and improve accessibility, then it is foreseeable that surgeons will need to be trained on robotic devices at an earlier stage of their careers.

The overwhelming majority of studies investigating the problem of skills transfer from laparoscopy to robotic surgery have utilized the Da Vinci Surgical System (Intuitive Surgical Inc.) because of its pioneering role in robot-assisted surgery. Recently, the Senhance Surgical System (TransEnterix Inc.) was approved by the FDA as a robot-assisted surgical device for colorectal surgery, cholecystectomy, inguinal hernia repair, and gynecologic surgery in adults [9]. Unlike the Da Vinci, the Senhance uses non-wristed instruments that mimic laparoscopy, and it has therefore been marketed as a robot-assisted digital laparoscopy device [10]. The question of whether previous laparoscopy training can enhance or accelerate the transition to robot-assisted digital laparoscopy is not known and warrants investigation for novice as well as experienced trainees.

The aim of the study was to evaluate the first-time robotic surgery experience of naive learners after first exposing them to laparoscopy. To our knowledge, this is the first study which explores how laparoscopy training affects initial performance on a robot-assisted digital laparoscopy device.

## Materials and methods

Our null hypothesis was that there would be no difference in the measured objective performance and subjective experience of naive learners who had been exposed to laparoscopy and evaluated on robot-assisted digital laparoscopy outcomes. This would be the case if basic psychomotor skills do not readily transfer from laparoscopy (study exposure) to robot-assisted digital laparoscopy (study outcome).

To determine the minimum sample size needed to test our hypothesis, we conducted an a priori power analysis using G*Power 3.1.9.4 [11]. We calculated effect sizes (t-test matched pairs, *d* = 1.73–2.01) from previous learning studies where novice medical students completed basic manipulation tasks, demonstrating 90% of their learning potential after approximately six repetitions, equivalent to about 20 min of laparoscopy training [12, 13]. Based on this, we calculated that we would need at least six participants per independent group to achieve adequate statistical power, defined as > 80%.

**Fig. 1:**
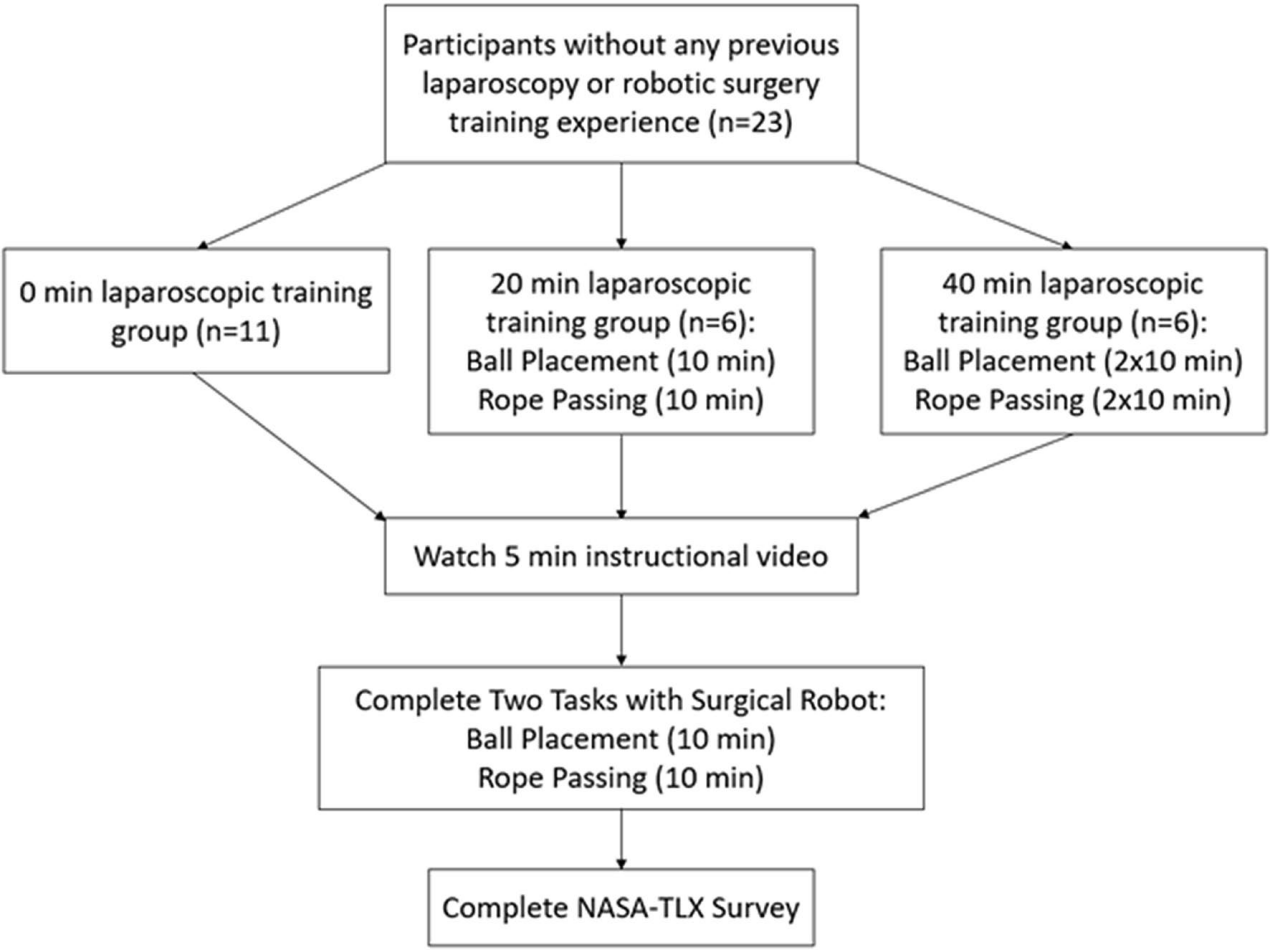
Study protocol

Medical students (*n* = 23) without prior training in laparoscopy and robotic surgery self-enrolled through an email self-scheduler which was sent to the English medicine programs at Jagiellonian University Medical College. A flow-chart depicting the study protocol in our study is shown in Fig. 1.

**Fig. 2:**
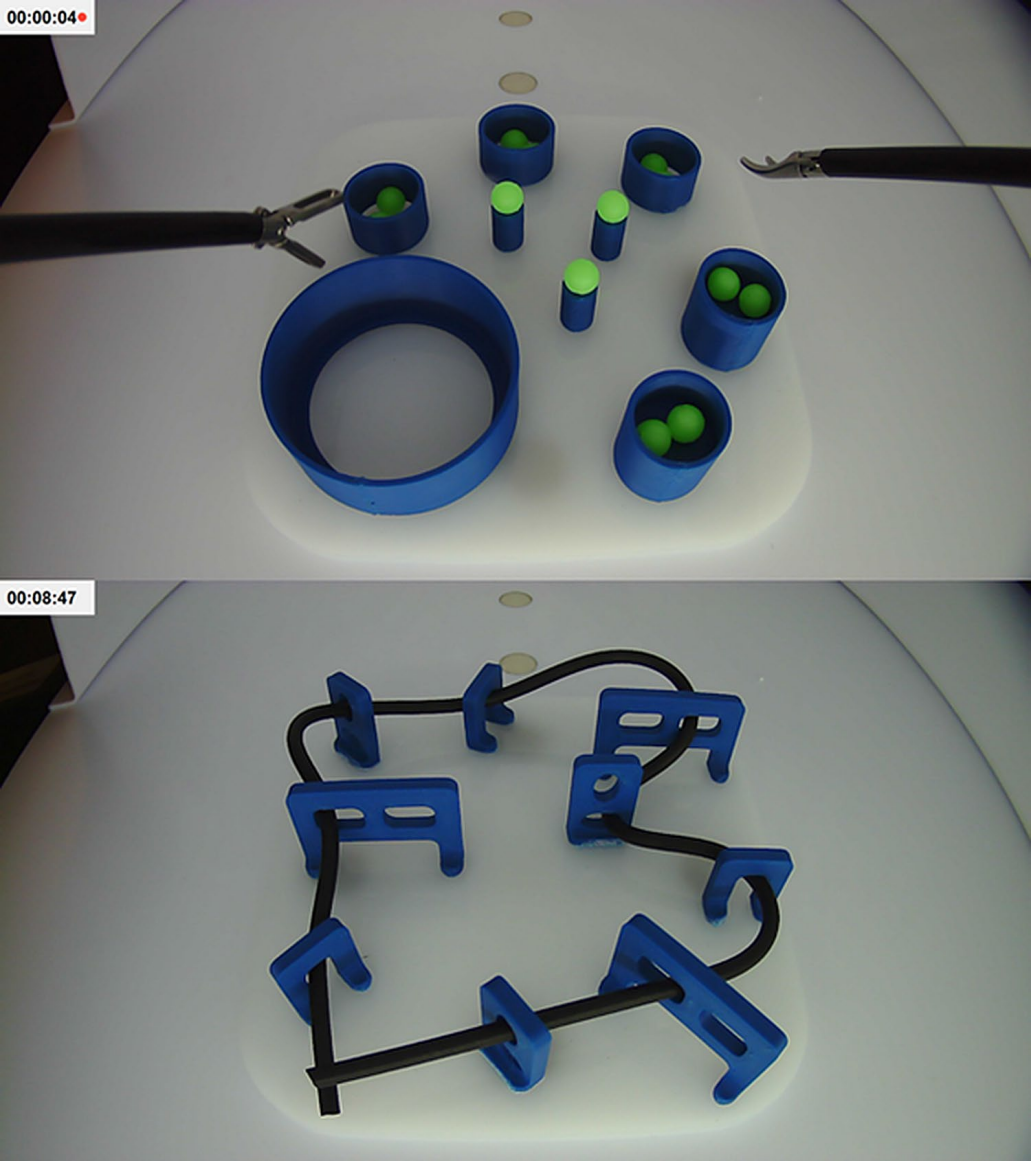
Laparoscopy training tasks and suggested completion patterns for ball placement and rope passing

The Laparo Advance (Laparo LLC, Wroclaw, Poland) trainer was used for laparoscopy task training, as shown in Fig. 2. Participants were instructed to complete the ball placement task as many times as possible within 10 min, alternating between left and right hand to place each ball. After 10 min, the participants switched to the rope passing task, using both hands, repeating as many times as possible for another 10 min. The 0 min training group (*n* = 11) served as the control and did not complete laparoscopy training. The 20 min training group (*n* = 6) completed both tasks, 10 min each. The 40 min training group (*n* = 6) completed both tasks twice, alternating tasks every 10 min.

The robot-assisted tasks were completed with the Senhance Surgical Robotic System (TransEnterix Inc., Morrisville, NC) and the Kroton LLC (Warsaw, Poland) trainer box. For simplicity, eye-tracking was disabled, but haptic feedback was left intact. The robot was introduced to the students using a 5 min instructional video, which was filmed by us and uploaded to YouTube [14]. Subjects were verbally instructed to “pay attention to the goals of the task, use both hands, remember the clutch”, and that “the experiment and clock may be paused if there are technical interruptions such as when a robot arm moves out of range, becomes stuck or stops responding”. During interruptions, the clock was paused, the robot arms were reset to the neutral starting position, and the clock was resumed. No clues were given to participants on how to best perform a task. The time limit for each task was 10 min. Videos of performance were recorded and later evaluated by study investigators for task completion, time, clutch use, and errors including number of ball drops, number of instances where the instruments moved out of view, and number of interruptions requiring manual adjustments. Subjects completed the NASA-TLX questionnaire immediately after the robotic study, which is a multi-dimensional survey used to evaluate workload and task demand [15].

To establish performance benchmarks, the senior study investigator who operates with laparoscopy, the Da Vinci System, and the Senhance System completed the ball placement and rope passing tasks three times each, followed immediately by the NASA-TLX survey.

Statistical analysis was performed on SPSS version 26 (IBM Corp., Armonk, NY). The Pearson Chi square test was used to compare completion rates. For the remaining performance measures, which are non-categorical, non-parametric tests were used without assumption of normality. The Independent-Samples Kruskal–Wallis Test was used for hypothesis testing between the 0 min (*n* = 11), 20 min (*n* = 6), and 40 min (*n* = 6) training groups. Correlations between performance variables were analyzed using Pearson Linear Regression and Spearman’s Rho. Significance level was 0.05 for all tests, except for correlations where it was 0.01. Plots were made using MATLAB 2019b (MathWorks Inc., Natick, MA).

## Results

### Ball placement and rope passing tasks—objective results

Results obtained from the study for the 0 min (*n* = 11), 20 min (*n* = 6), and 40 min groups (*n* = 6) are summarized in Table 1. Completion rate for the ball placement task was 10/11, 5/6, and 5/6, respectively (*p* > 0.05). Time spent on the task, balls placed and balls dropped, and clutch use did not differ significantly (*p* > 0.05). The number of subjects whose instruments moved outside the field of view was 2/11, 0/6, and 0/6 for the three groups. Meanwhile, 3/11, 0/6, and 1/6 subjects experienced interruptions requiring a pause of the experiment and readjustment to the starting position. Differences in the number of out-of-view instrument errors and instances requiring manual readjustment were not statistically significant (*p* > 0.05). Participants in these groups pressed the clutch pedal 16.4 ± 11.4, 18.3 ± 12.3, and 6.5 ± 5.4 times (*p* > 0.05). For reference, the expert completed the ball placement task without any errors or interruptions in 43.3 ± 3.1 s and used the clutch 2.0 ± 1.0 times (mean ± standard deviation of three attempts).

**Table 1:** Results of medical student performance on robot-assisted tasks

Mean ± SD	0 min group (*n* = 11)	20 min group (*n* = 6)	40 min group (*n* = 6)	*p* value
Total Time (s)	834.09 ± 249.74	889.33 ± 164.01	780.67 ± 319.61	0.77
NASA-TLX score	10.03 ± 2.21	10.68 ± 1.44	11.72 ± 1.92	0.24
Task 1: Ball placement				
Time (s)	345.18 ± 148.04	336 ± 165.82	285.5 ± 191.46	0.65
Instruments out of view	0.27 ± 0.65	0.00 ± 0.00	0.00 ± 0.00	0.32
Manual adjustments	0.36 ± 0.67	0.00 ± 0.00	0.33 ± 0.82	0.41
Ball drops	1.46 ± 1.21	2.00 ± 1.41	2.50 ± 2.43	0.66
Balls placed	3.73 ± 0.65	3.83 ± 0.41	3.67 ± 0.82	0.99
Clutch use	16.36 ± 11.39	18.33 ± 12.26	6.50 ± 5.43	0.052
Completion rate (%)	90.91	83.33	83.33	0.87
Task 2: Rope passing				
Time (s)	488.91 ± 180.99	553.33 ± 114.31	495.17 ± 165.46	0.55
Instruments out of view	0.45 ± 0.69	0.17 ± 0.41	1.00 ± 1.55	0.43
Manual adjustments	1.09 ± 1.22	1.00 ± 2.00	1.67 ± 2.25	0.55
Loops threaded	1.36 ± 1.36	1.00 ± 1.26	2.17 ± 0.98	0.23
Clutch use	31.36 ± 33.49	20.50 ± 16.13	10 ± 6.93	0.20
Completion rate (%)	36.36	16.67	50	0.47

For the rope passing task, completion rates for the 0 min, 20 min, and 40 min training groups were 4/11, 1/6, and 3/6 (*p* > 0.05), respectively. Time spent on the task and number of loops threaded did not differ significantly (*p* > 0.05). Within these groups, 7/11, 2/6, and 4/6 subjects experienced interruptions requiring a pause of the experiment (*p* > 0.05). Mean clutch use was 31.4 ± 33.5, 20.5 ± 16.1, and 10.0 ± 6.9, respectively (*p* > 0.05). For reference, the expert completed the rope passing task without errors in 40.0 ± 7.5 s and pressed the clutch 2.0 ± 1.0 times (mean ± standard deviation of three attempts).

**Fig. 3:**
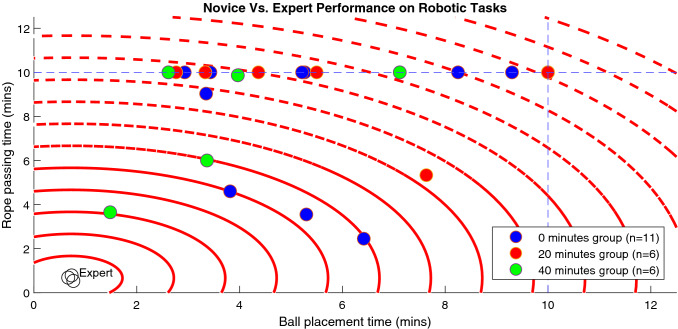
Scatter plot of time performance for study participants, with red circles marking 1 min intervals from the mean of expert times, and dashed blue lines marking the time limit for each task

A plot containing ball placement task time (x-axis) and rope passing task time (y-axis) for all participants including the expert is shown in Fig. 3. In our study, 15/23 subjects failed to complete the rope passing task within the time allowed, and within this group, 3/15 also failed to complete the ball placement task. In comparison, all subjects who completed the rope passing task also completed the ball placement task. The best performer in our study had task times that were within 3 min of the expert mean.

### NASA-TLX survey—subjective results

**Fig. 4:**
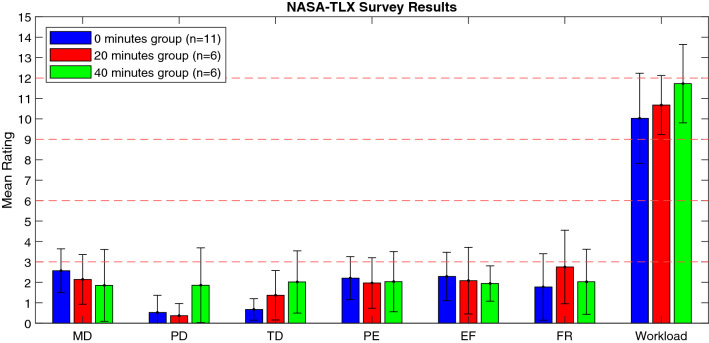
Bar graph of NASA-TLX scores for subjects, where *MD* mental demand, *PD* physical demand, *TD* temporal demand, *PE* performance, *EF* effort, and *FR* frustration

Survey results with relative contributions of Mental Demand (MD), Physical Demand (PD), Temporal Demand (TD), Performance (PE), Effort (EF), and Frustration (FR) are displayed in Fig. 4. Subjects in the 0 min, 20 min, and 40 min groups reported workloads of 10.03 ± 2.21, 10.68 ± 1.32, and 11.73 ± 1.75 out of 15, respectively. However, increased workload with training was not statistically significant (*p* > 0.05). For comparison, the expert reported a workload score of only 0.75 out of 15.

### Regression analysis

Pearson’s and Spearman’s tests did not reveal statistically significant correlations between laparoscopy training time (0 min, 20 min, and 40 min) and the measurements listed in Table 1 (*p* > 0.01). NASA-TLX score was moderately correlated with total time spent on tasks (*r* = 0.623, rho = 0.681, *p* < 0.01).

## Discussion

The time to achieve mastery in basic and advanced robotic tasks in a simulator setting has been estimated at 10 h for junior and senior surgery residents, respectively [16]. However, residents from institutions where structured curricula are not mandatory may fail to complete robotic surgery training, citing barriers such as lack of time, lack of access to robot or simulators, and lack of motivation, in contrary to overwhelming initial interest to gain fundamental robotic skills [17]. Laparoscopy education, notably the Fundamentals of Laparoscopy Surgery (FLS) curriculum, is more accessible to surgery residents, but evidence is lacking as to whether these skills transfer to robot-assisted surgery.

Learning a basic laparoscopy task, such as the FLS peg transfer, follows an inverse learning curve, with 90% of gains occurring in the first 5.8 ± 2.3 trials for a group of 16 medical students [13]. In another study of 16 new surgical residents, peg transfer task times of 201 ± 84 s were measured prior to FLS training [18]. If 90% of gains occur in the first 5.8 ± 2.3 repetitions for this task, then a large effect size should be measurable after a training time of 20 min, which should allow for six repetitions of this task.

We selected the ball placement and rope passing tasks for our study because they satisfied the same psychomotor criteria as the FLS peg transfer task, namely depth perception, visual-spatial perception in a 2D setting, and the coordination of dominant and non-dominant hands, which are important skills for suturing and needle positioning [19]. The maximum time limit allowed for this foundation level task during FLS curriculum testing is 300 s, with a proficiency benchmark of 48 s [20]. For comparison, the robot-assisted version of the ball placement and rope passing tasks used in our study was 600 s, with measured proficiency benchmarks of 43.3 ± 3.1 and 40.0 ± 7.5 s, respectively.

We hypothesized that an effect size would be measurable if laparoscopy learning had occurred, and if these skills then transferred to the robot-assisted digital laparoscopy platform. However, we found no statistically significant differences in objective measures, such as time spent on tasks, completion rate, clutch use, out of view instruments, errors, and manual adjustments.

We also measured subjective differences related to the performance of a task which may not be reflected in objective measures, such as when two or more groups achieve similar performance times despite one group that finds the task to be significantly more difficult than the other groups. NASA-TLX workload was greater in laparoscopy-exposed groups in our study, but this result was not statistically significant. This observation could be due to ergonomic differences between laparoscopy and robotic surgery [21].

Altogether, our study results suggest that laparoscopy exposure, in the form of limited psychomotor skills training, does not affect initial robot-assisted surgery performance among learners. This study supports the idea that training in robotic surgery ought to take place in a robot-assisted simulation environment.

With respect to study limitations, there was self-selection bias in our study because participants self-enrolled and self-assigned into their own groups. The number of participants in our study was also small, affecting the precision of our results. Practical limitations with respect to operating room time, student availability, surgeon availability, robot availability, and experiment costs make it difficult to organize larger and more comprehensive studies.

Although randomization and blinding could be achieved by performing this study on a virtual reality simulator, simulators are not yet available for the Senhance Surgical System. Simulation is also not the same as real-world surgery in an operating room setting, where technical difficulties and instrument errors can occur, as demonstrated by our study. Additionally, the presence of an attending surgeon could have influenced our experiment, but trainees at most institutions (including ours) are not allowed to operate without direct supervision. Interruptions are stressful and can influence the subjective experience of users, which our study aimed to capture. These arguments support and strengthen the validity of our study.

Several studies have previously reported faster learning curves and improved retention of skills with robotic assistance as compared to laparoscopy [22–25]. With respect to basic manipulation tasks, improved task speeds with robot assistance have been measured as compared to laparoscopy, but with minimal transfer effects [26]. Among studies looking at skills transfer, one study compared novices completing a ball drop task with laparoscopy or the RoSS simulator, and found that while both groups improved after training, the degree of improvement was equal, indicating there is no skills transfer [27]. Other studies have argued that skills transfer effects from laparoscopy to robotic surgery may be more pronounced with difficult tasks, such as suturing [22, 23]. In our view, laparoscopy and robotic surgery are different domains, perhaps requiring different skills. Previously, novice users have demonstrated rapid adaptation to the Senhance device, regardless of experience level [28]. Validated robotic surgery curricula, such as the Fundamentals of Robotic Surgery (FRS), offer a direct and efficient path for novices to become proficient in robotic skills without embarking on the intermediate step of laparoscopy [29].

Individual performance was highly variable, and it would have been worthwhile to ask participants about their backgrounds and career goals to investigate factors differentiating high and low performers. One study has found evidence to suggest that objective innate ability may distinguish students interested in surgical careers from others [30]. However, it must be remembered that innate or initial ability may not correlate with the rate of improvement of robotic skills, which requires practice.

## Conclusion

Limited laparoscopy exposure may not improve the initial performance of novices on the Senhance Surgical System, a robot-assisted digital laparoscopy device. Further investigation is needed to determine how laparoscopy training affects robot-assisted surgery performance.

## Data Availability

Available upon request.

## Code availability

Available upon request.
